# Experimental electronic structures of copper complexes with a bi­phenyldi­imino di­thio­ether – a model for blue copper proteins

**DOI:** 10.1107/S2052252524012107

**Published:** 2025-01-30

**Authors:** Marek Fronc, Martin Breza, Lukáš Bučinský, Ingrid Jelemenská, Jozef Kožíšek

**Affiliations:** a Institute of Physical Chemistry and Chemical Physics Slovak University of Technology in Bratislava Radlinského 9 Bratislava SK-81237, 81237 Slovakia; Université de Sherbrooke, Canada

**Keywords:** electronic structures, electron density distributions, charge densities, blue copper proteins, quantum crystallography, high-resolution X-ray reflection data, bi­phenyldi­imino di­thio­ether (*bite*) ligands

## Abstract

The experimental electron density distributions in two coordination compounds – one with a central Cu(I) atom and the other with Cu(II), coordinated by the same bi­phenyldi­imino di­thio­ether (*bite*) type of ligand – have been obtained from high-resolution X-ray reflection data to model the possible electron predisposition for the redox reaction in blue copper proteins. The *bite* ligand has been adapted to the conformation required by the central atom.

## Abstract 2

Differences in the electronic structures of Cu(I) and Cu(II) coordination compounds with the same ligand are studied. These compounds act as a model for blue copper proteins.

## 1. Introduction

Copper is a first-row transition metal that is essential for life, it is present in almost all living organisms. Copper is mostly present in compounds in its formal oxidation states +I and +II. There are several types of proteins that contain copper in their ‘prosthetic group’ (Malmström, 1982 ▸). The so-called blue copper proteins contain a type 1 copper ion (characterized by their EPR spectra) and are distinguished by a strong absorption band around 600 nm, giving them their deep-blue color. The inner coordination sphere of the type 1 Cu sites mostly consists of two Cu(II)—N(His) bonds, one Cu(II)—S(Cys) bond and one Cu(II)—S(Met) bond (Arcos-López *et al.*, 2020 ▸). They are small and generally soluble proteins involved in monoelectron transfer processes in biological systems. Like the central iron atom in hemoglobin, the activation of oxygen in the blue protein is associated with a change in the oxidation state of the central copper atom from Cu(II) to Cu(I). The theoretical study of model structures of blue copper proteins accelerated after the determination of the crystal structure of azurine (Baker, 1988 ▸; Chen *et al.*, 1998 ▸). Proteins themselves are not well suited for experimental studies of their electronic structure, especially because of the size of the molecule. Instead, smaller molecules such as Flanagan’s copper(I) (**A**) and copper(II) (**B**) complexes (Flanagan *et al.*, 1997 ▸) seem to be suitable models for such a study. The central copper atom in both complexes is coordinated by the same bi­phenyldi­imino di­thio­ether ligand but, of course, in a different conformation. In **A**, the central atom is pseudo-tetrahedrally coordinated by two sulfur and two nitro­gen atoms of the ligand. The charge of the complex cation is eliminated by the counter BF_4_^−^ anion. In **B**, the coordination polyhedron is pseudo-octahedral in the equatorial plane with two sulfur atoms and two nitro­gen atoms in a *cis* arrangement. The axial positions of the coordination polyhedron are completed by the fluorine atoms of two neighboring BF_4_^−^ anions. This study is devoted to the comparison of the electron density distributions in these two complexes.

Herein we present multipole model (MM) electron density studies of **A** and **B**, which are compared with theoretical DFT results. The electronic structures are considered by means of the topology of the electron density, the atoms in molecules (AIM) approach and/or *d* orbital populations derived for the MM.

## 2. Experimental

### 2.1. Material and methods

#### 2.1.1. Synthesis and crystal growth

The complexes **A**, [Cu^I^(C_28_H_22_N_2_S_2_)]BF_4_, and **B**, [Cu^II^(C_28_H_22_N_2_S_2_)](BF_4_)_2_, were prepared according to the paper by Flanagan *et al.* (1997 ▸). All reagents were obtained from Sigma–Aldrich (p.a. grade) and solvents from mikroCHEM (p.a. grade). After crystallization, suitable crystals were selected for the X-ray diffraction experiments.

#### 2.1.2. Data collection

A high-quality yellow single crystal with the dimensions 0.115 × 0.115 × 0.310 mm for **A** and a black single crystal with the dimensions 0.048 × 0.146 × 0.195 mm for **B** were measured on an Eulerian four-circle Stoe STADIVARI diffractometer with a Dectris Pilatus 300 K detector and Incoatec IµS Ag microfocus source (Ag *K*α, λ = 0.56083 Å) at 100 K using a nitro­gen gas open-flow Cobra cooling system from Oxford Cryosystems. Two detector positions were used for 37 omega scans (2θ = 0.0 and 45.7°) with a 0.2° frame width and an exposure time of 12 and 120 s, respectively, for **A**, and 28 omega scans (2θ = −41.7°) with a 0.1° frame width and exposure time of 40 s for **B**. Data reduction was performed using *X-Area Integrate* (version 1.84.1) and *X-Area X-Red32* (version 1.65.0.0; Stoe & Cie, 2018 ▸). For absorption correction, a crystal-shaped model with measured faces was employed. Details of the X-ray diffraction experiment conditions and crystallographic data are given in Table 1 ▸.

We tried to find the explanation for the low goodness-of-fit (GooF) of 0.701 for **A** in the multipole refinement. The explanation was found in the *SHELXL* IAM refinement. The reflections above sin θ/λ > 1.0 Å^−1^ were underestimated [Figs. S2(*a*) and S2(*b*) of the supporting information]. For **B** there is a similar effect. For the *SHELXL* refinement GooF = 0.725 and for *XD2016* GooF = 1.476. These differences could be explained by overestimating the reflections above sin θ/λ > 1.0 Å^−1^ [Figs. S2(*c*) and S2(*d*)].

#### 2.1.3. Electron density refinements

**Figure 1:**
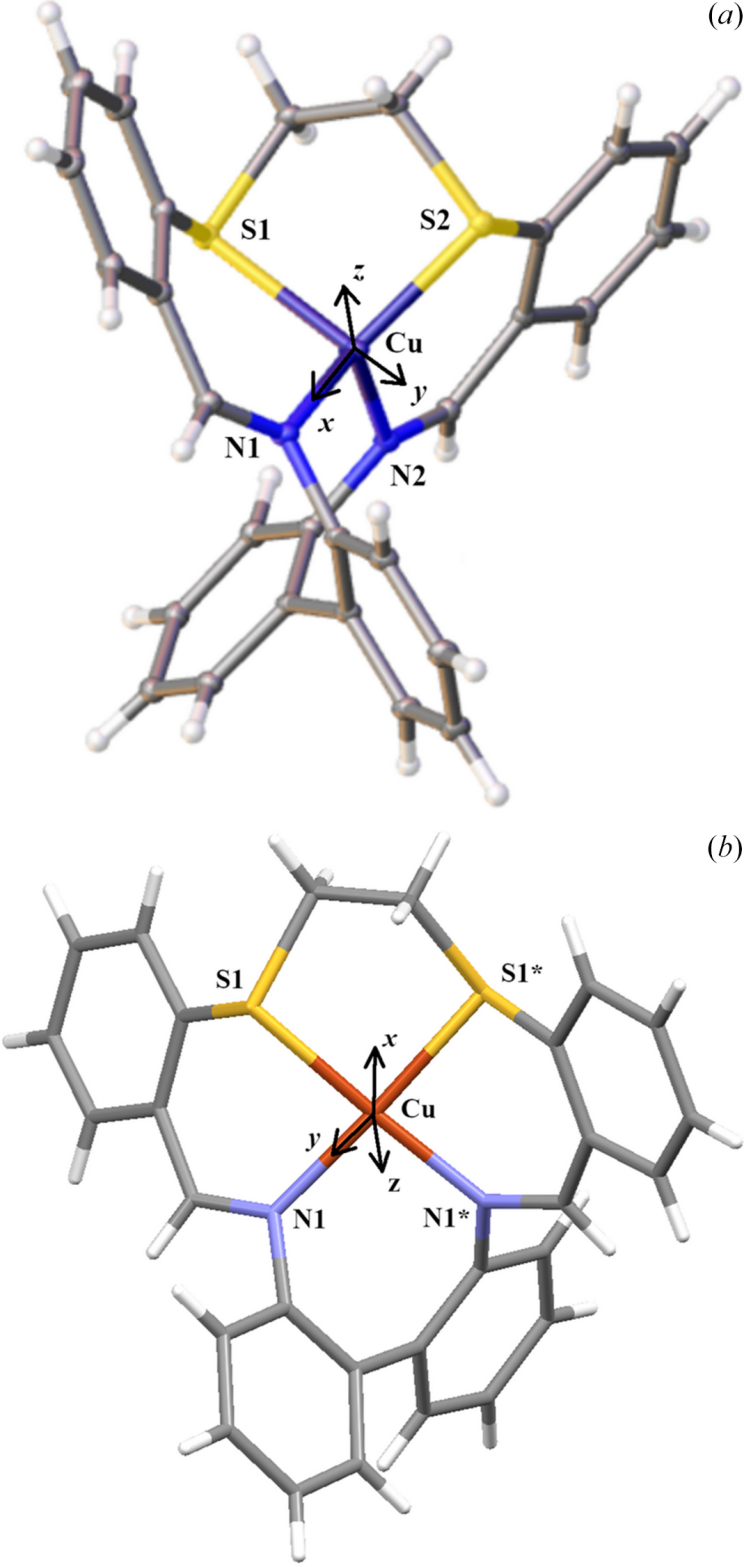
Definition of local coordinates for **A** and **B**. (*a*) Local coordinates for **A**. *x*: Cu—N1, *y*: in the Cu—N1—N2 plane. The *z* axis is perpendicular to the *xy* plane. (*b*) Local coordinates for **B**. *x*: Cu—DUM1, *y*: in the Cu—DUM1—N1 plane. The *z* axis is perpendicular to the *xy* plane

The structure was solved by the dual-space algorithm as implemented in *SHELXT* (Sheldrick, 2015 ▸). The IAM was refined using *SHELXL* (Sheldrick, 2015 ▸) utilizing the graphical user interface *Olex2* (Dolomanov *et al.*, 2009 ▸). For MM refinement, the Hansen–Coppens model (Hansen & Coppens, 1978 ▸) was used. Starting atom coordinates and atom displacement parameters (ADPs) were taken from the routine *SHELXL* refinement, and all other refinements were carried out on *F*^2^ using the *XD2016* suite of programs (Volkov *et al.*, 2016 ▸). Our refinement strategy was the same as described in our previous studies (Kožíšek *et al.*, 2002 ▸; Herich *et al.*, 2018 ▸; Adamko-Kožíšková *et al.*, 2021 ▸), including the use of the relativistic Su and Coppens wavefunctions (Su & Coppens, 1998 ▸). For compounds **A** and **B**, the scattering curves of the Cu^+^ cation ([Ar] 4s^1^3d^9^) and the Cu^2+^ cation ([Ar] 4s^0^3d^9^) were used, respectively. In Kappa and in unrestricted refinements, the charges on cations and anions were constrained to integer values. The local coordinate system for the copper atoms was chosen so that the *x* axis was pointing to the nearest bonded atom N1, the *xy* plane contained the atom N2 in the case of **A** and the *z* axis was pointing to a DUMMY atom (1/2, 3/4, 0.4). The *zy* plane contains the atom N1 in the case of **B** to ensure the twofold local symmetry for the central Cu atom [as shown in Figs. 1 ▸(*a*) and 1 ▸(*b*)]. Hexadecapoles for Cu and S; octapoles for F, N, C and B; and dipoles for H were used in the MM. Constraints due to the local symmetry [except for the copper atom in the center of symmetry due to the twofold axis parallel to the local *z* axis in **B**] as well as chemical constraints were not used. In the case of charge rearrangements between individual atoms (Kappa refinement and unrestricted multipole refinement), the cation and anion were refined separately. Kappa prime was not refined. We also attempted to apply an anisotropic secondary extinction correction (Herich *et al.*, 2018 ▸), but in both crystal structures this correction was found to be unnecessary. AIM analysis was done using *XDPROP* from the *XD2016* suite of programs.

#### 2.1.4. Quantum-chemical calculations

The electronic structures of the [Cu(*bite*)]^+^ complexes of **A** in the singlet ground state and [Cu(*bite*)]^2+^ of **B** in the doublet ground state were investigated using the M06 hybrid functional (Zhao & Truhlar, 2008 ▸) with GD3 dispersion correction (Grimme *et al.*, 2010 ▸) and cc-pVTZ basis sets for all atoms from the Gaussian library (Frisch *et al.*, 2013 ▸). Optimized structures of the above complexes were checked on imaginary vibrations (no negative frequencies). Natural population analysis (Reed *et al.*, 1985 ▸) was used to evaluate *d*-electron populations at the Cu atoms. All quantum-chemical calculations were performed using the *Gaussian09* software (Frisch *et al.*, 2013 ▸). The electron structures of the complexes under study were evaluated in terms of the quantum theory of atoms in molecules (QTAIM) (Bader, 1990 ▸) such as atomic charges (obtained by integration over atomic basins up to 0.001 a.u.) for atoms and electron density, its Laplacian, and ellipticity at bond critical points (BCPs) for bonds using the *AIMAll* software (Keith, 2017 ▸). The overlap of the complexes studied was visualized with the *PyMol* package (Schrodinger, 2015 ▸).

## 3. Results and discussion

### 3.1. Structure description

The crystal structure of **A** consists of a [Cu(*bite*)]^+^ cation (*bite* = a bi­phenyldi­imino di­thio­ether type ligand, more exactly 15,18-di­thia-9,24-diaza­tetra­benzo­[*a*,*e*,*g*,*k*]­cyclo­hexa­deca-9,23-diene) and a [BF_4_]^−^ anion. The coordination polyhedron of Cu(I) in **A** is a deformed tetrahedron [Figs. 1 ▸(*a*) and S1(*a*)] with bonding distances Cu—N1, Cu—N2, Cu—S2 and Cu—S1 of 1.9406 (6), 1.9609 (5), 2.19414 (8) and 2.3264 (1) Å, respectively, and with the *X*—Cu—*X* (*X* = S, N) angle in the interval 97.44 (2)–132.99 (2)° (see Table 2 ▸ and Table S1 of the supporting information). The difference in Cu—S bond lengths in **B** is similar to that in azurine (Chen *et al.*, 1998 ▸). The crystal structure of **B** consists of a [Cu(*bite*)]^2+^ cation and two [BF_4_]^−^ anions. The copper atom is in a special position, so the independent part of the cation is its half. The central atom Cu(II) in **B** is coordinated by the same ligand as Cu(I) in **A** with two nitro­gen and two sulfur atoms in the equatorial plane of the pseudo-octahedral polyhedron in a *cis* arrangement. The axial positions are completed by a fluorine atom of the [BF_4_]^−^ anion [Figs. 1 ▸(*b*) and S1(*b*)]. Due to symmetry, there is only one value for the Cu—S1 bond distance [2.2915 (2) Å], for Cu—N1 [1.9900 (9) Å] and likewise for Cu—F1 [2.530 (2) Å] (see Table 2 ▸). The *X*—Cu—*X* (*X* = S, N) angle is in the range 87.50 (4)–176.69 (4)° (see Tables 2 ▸ and S1). The interactions between the anion and cation for both structures will be discussed later.

An error analysis shows the following results (in the supporting information). The residual density calculated by fast Fourier synthesis (XDFFT) for all diffractions for **A** is 0.36 e Å^−3^ at 0.71 Å from the sulfur atom S2 and −0.33 e Å^−3^ at 0.57 Å from the nitro­gen atom N2 with a mean value of 0.068 e Å^−3^, and for **B** is 0.25 e Å^−3^ at 0.02 Å from the copper atom Cu and −0.25 e Å^−3^ at 0.44 Å from the sulfur atom S1 with a mean value of 0.058 e Å^−3^. Residual maps are given in Figs. S2(*a*) and S2(*b*).

The dependence of the scale factor on sin θ/λ for **B** (Abrahams & Keve, 1971 ▸; Farrugia, 2012 ▸) shows a problem for high-order reflections above sin θ/λ = 0.7 Å^−1^ [Figs. S3(*a*) and S3(*b*)]. We repeated the multipole refinement with a trim to *d*_min_ = 0.70 Å [Fig. S3(*c*)]. The residual density (trim data for **B**) is 0.34 e Å^−3^ at 0.88 Å from the sulfur atom S1 and −0.30 e Å^−3^ at 0.87 Å from the carbon atom C10 with a mean value of 0.069 e Å^−3^ [Fig. S2(*c*)].

The same situation was observed in a fractal plot of the residual density (Meindl & Henn, 2008 ▸). The plot has a symmetrical shape for the entire sin θ/λ range of the dataset for **A** with ρ_min_ = −0.09 e Å^−3^and ρ_max_ = 0.09 e Å^−3^, and for **B** with ρ_min_ = −0.22 e Å^−3^ and ρ_max_ = 0.22 e Å^−3^, or ρ_min_ = −0.13 e Å^−3^ and ρ_max_ = 0.13 e Å^−3^, respectively [see Figs. S4(*a*)–S4(*c*)].

We tried to find the explanation for the low GooF of 0.701 for **A** in the multipole refinement. The explanation was found in the *SHELXL* IAM refinement. The reflections above sin θ/λ > 1.0 Å^−1^ were underestimated [Figs. S3(*a*) and S3(*b*)]. For **B** there is a similar effect. For the *SHELXL* refinement, GooF = 0.725 and for *XD2016* GooF = 1.476. These differences could be explained by overestimating the reflections above sin θ/λ > 1.0 Å^−1^ [Figs. S3(*c*) and S3(*d*)]. We compared the *SHELXL* refinement of the full dataset with the trimmed data (Table S1) and AIM analysis after the *XD2016* refinement (Table S2) and there are no important differences. Low-order reflections (sin θ/λ < 0.7 Å^−1^) introduce enormous noise and do not contribute significantly to the static electron density.

### 3.2. AIM analysis

The electronic structure of the compound under study was investigated using an AIM topological analysis of the electron density (Bader, 1990 ▸). The results were evaluated in terms of atomic charges obtained using the electron density integrated over atomic basins and the bond characteristics in terms of the electron density ρ at BCPs corresponding to saddle points at bond paths between individual atoms, its Laplacian (∇^2^ρ) and bond ellipticity (ε). The BCP electron density, ρ_BCP_, is proportional to the bond strength; the value and sign of its Laplacian, ∇^2^ρ_BCP_, describe the relative electron density contribution of the bonded atoms to the bond (covalent *versus * dative bonding); its bond ellipticity, ε, describes its deviation from cylindrical symmetry (such as in ideal single or triple bonds) due to its double-bond character, mechanical strain and/or other perturbations. The main differences between the Cu(I) (**A**) and Cu(II) (**B**) coordination cations are in the type of coordination polyhedron, as discussed previously in Section 3.1. The results of the AIM analysis [see Tables 3 ▸, 4 ▸, S2(*a*), S2(*b*) and S2(*c*)] and the charge of the central atom (see Table 5 ▸) allow us to comment further on the strength of the coordination bonds. In both compounds the Cu—N bonds are stronger than the Cu—S bonds. The interatomic distances in **A** and **B** for Cu—N1, Cu—N2 and Cu—N1 [1.9406 (6), 1.9609 (5) and 1.9855 (12) Å, respectively], as well as for Cu—S1, Cu—S2 and Cu—S1 [2.3264 (1), 2.1941 (1) and 2.2922 (4) Å, respectively] (see Table 2 ▸) agree with the values of the electron density at the BCP (see Table 3 ▸). The same trend applies to its Laplacians (∇^2^ρ_BCP_) for Cu—N1, Cu—N2 and Cu—N1 with values of 0.691 (5), 0.671 (4) and 0.671 (6) e Å^−5^, respectively; for Cu—S1, Cu—S2 and Cu—S1 with values of 0.442 (3), 0.598 (4) and 0.511 (4) e Å^−5^, respectively; ∇^2^ρ_BCP_ for Cu—N1, Cu—N2 and Cu—N1 with values of 11.257 (7), 10.603 (6) and 9.943 (8) e Å^−5^, respectively; and for Cu—S1, Cu—S2 and Cu—S1 with values of 5.329 (3), 7.024 (4) and 6.075 (4) e Å^−5^, respectively (see Table 3 ▸). The average values of the electron density and the Laplacian in the BCP for the Cu—S bond in **B** are similar to those for **A**. Interesting results could be seen when comparing the BCP electron density and its Laplacian from experiment and DFT in X-ray and optimized geometries. The trends in the experimental and DFT results in X-ray geometries are consistent, but opposite to the trends in the experimental and DFT results in optimized geometries [see Tables 3 ▸, S2(*b*) and S2(*c*)]. This may be due to the non-bonding interactions with neighboring molecules that are not included in the DFT studies (see Table 3 ▸). Experimentally (MM) derived charges on specific atoms mainly indicate differences in the copper atoms (see Table 5 ▸). The charges on the central atoms are always lower compared with the formal oxidation state (Herich *et al.*, 2018 ▸; Kožíšek *et al.*, 2004 ▸, 2021 ▸; Scatena *et al.*, 2019 ▸, 2020 ▸). Although the difference between the formal oxidation states in **B** and **A** is one electron, the AIM charge difference calculated within the copper atomic basins is only 0.49 e. If the *bite* ligand is divided into S and N parts, the S part in **B** [S parts for **B**: S1, C1–C7, C14, H4–H7, H14A, H14B] is about +0.18 e more positive than in **A** and the N part in **B** is about −0.45 e more negative than in **A**. The difference |0.18| + |−0.45| = 0.63 compared with 0.49 may be due to experimental error. Interestingly, the charge on the sulfur atoms in **A** (+0.21 and +0.25) is more positive compared with +0.06 in **B**.

An interesting situation is shown by the comparison of the experimental and the theoretical charges of the atoms (see Table 5 ▸). The trends in charges for the copper atom, the nitro­gen atoms and the carbon atoms bound to a nitro­gen atom are in good agreement, sulfur atoms and carbon atoms bound to a sulfur atom have more positive experimental charges than the corresponding theoretical ones. The theoretical charges of all other atoms do not reflect the trend of experimental charges and are close to neutral values. As the DFT calculations are related to isolated model cations without any environmental influences, it can be supposed that the experimental electronic structure can better capture the redistribution of the valence electron density in the ligand for different oxidation states of the central atom and the non-identical coordination of the central atom by the ligand.

**Figure 2:**
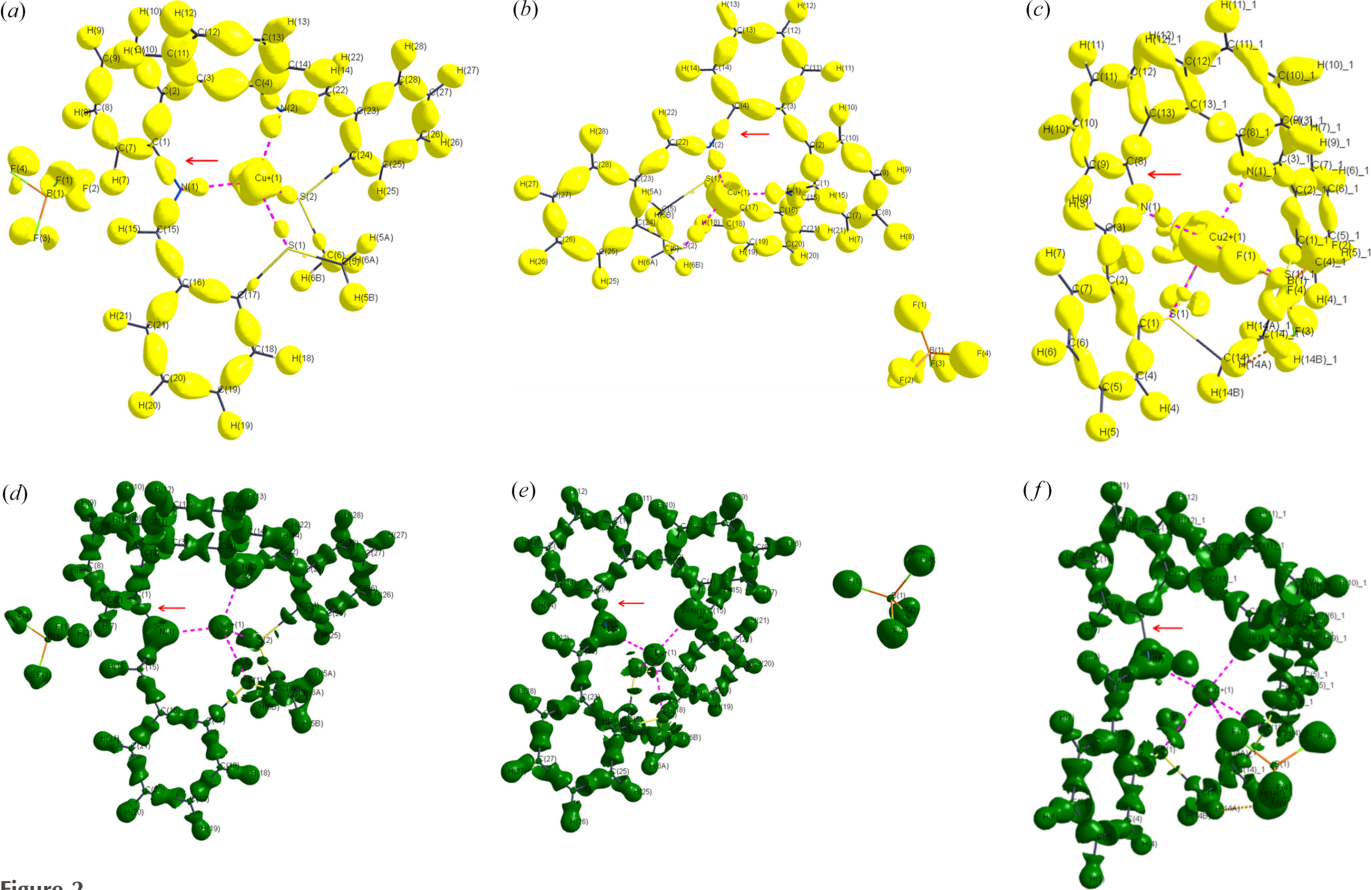
3D plots of the static deformation density and the Laplacian of the electron density in **A** and **B**. (*a*) 3D plot of the static deformation density of compound **A** at an isosurface value of 0.3 e Å^−3^ [see the N1—C1 region, red arrow]. (*b*) 3D plot of the static deformation density of compound **A** at an isosurface value of 0.3 e Å^−3^ [see the N2—C4 region, red arrow]. (*c*) 3D plot of the static deformation density of compound **A** at an isosurface value of 0.3 e Å^−3^ [see the N1—C8 region, red arrow]. (*d*) 3D plot of the Laplacian of the electron density at an isosurface value of 5 e Å^−5^ [see the N1—C1 region, red arrow]. (*e*) 3D plot of the Laplacian of the electron density at an isosurface value of 5 e Å^−5^ [see the N2—C4 region, red arrow]. (*f*) 3D plot of the Laplacian of the electron density at an isosurface value of 5 e Å^−5^ [see the N(1)—C(8) region, red arrow].

**Figure 4:**
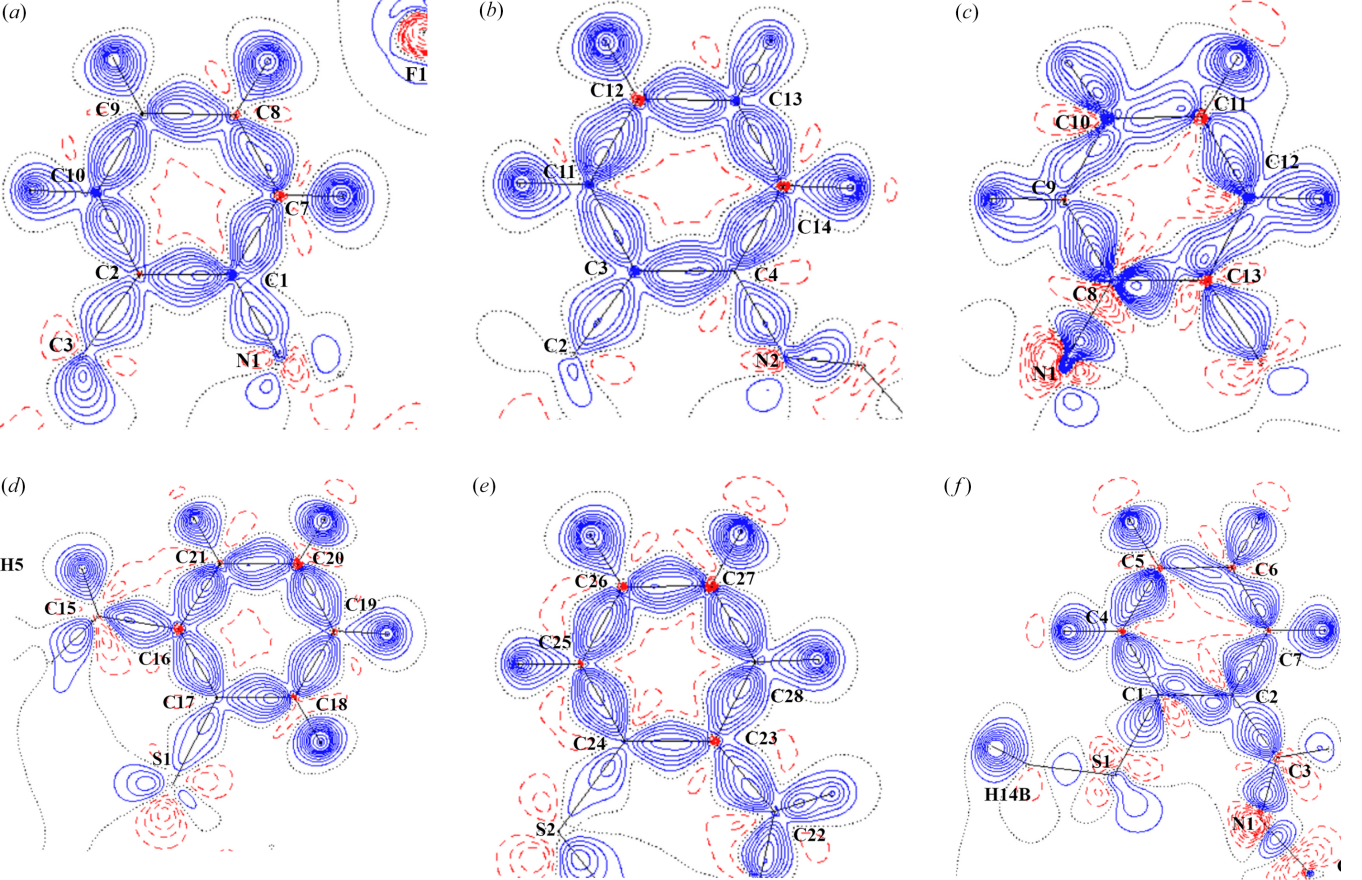
Static deformation density for **A** and **B**. The contour spacing is 0.10 e Å^−3^, with positive contours drawn with a solid blue line and negative contours with a dashed red line. (*a*) Static deformation density for **A**. Plane defined by the C2, C1 and C10 atoms. (*b*) Static deformation density for **A**. Plane defined by the C3, C4 and C11 atoms. (*c*) Static deformation density for **B**. Plane defined by the C8, C13 and C9 atoms. (*d*) Static deformation density for **A**. Plane defined by the C16, C17 and C21 atoms. (*e*) Static deformation density for (**A**). Plane defined by the C24, C23 and C25 atoms. (*f*) Static deformation density for **B**. Plane defined by the C1, C2 and C4 atoms.

Notable results were found for the nitro­gen–carbon bonds [N1—C1 and N2—C4 in **A**, and N1—C8 in **B**]. The largest differences in charges found for the nitro­gen atoms [N1 and N2 are −0.80 and −0.99 for **A**, respectively, and −1.50 for N1 in **B**; see Table 5 ▸]. Relatively high negative charge on the N1 nitro­gen atom for the Cu(II) complex (**B**) results in polarization of the electron density on the N1 atom and depletion of the electron density mediating the N1—C8 covalent bond [Figs. 2 ▸(*a*)–2 ▸(*f*) and 3 ▸(*a*)–3 ▸(*d*)]. The N1—C8 electron density in the BCP of 1.38 e Å^−3^ for **B** is lower than the N1—C1 and N2—C4 ones of 1.95 and 1.97 e Å^−3^, respectively, for **A** [see Table S2(*a*)]. The DFT results do not show the same effect [see Tables S2(*a*)–S2(*c*)]. It could be said that while in **A** the nitro­gen–carbon bond is covalent according to the BCP Laplacian value (−11.70 and −17.15 e Å^−5^ for N1—C1 and N2—C4, respectively), in **B** the corresponding bond can be designated as a non-bonding interaction for the MM electron density topology, or at least not a typical covalent bond (7.50 e Å^−5^ for N1—C8). Since a part of the MM electron density in the phenyl group is located both above and below the ring plane, the carbon atom has a depleted electron density in the direction of the N—C bond, similar to a ‘σ-hole’ in halogen–halogen bonds (Nayak *et al.*, 2009 ▸) or in a tetrel bond [Figs. 2 ▸(*a*)–2 ▸(*f*), 3 ▸(*a*)–3 ▸(*d*) (Bauzá *et al.*, 2013 ▸; Niu *et al.*, 2023 ▸; Hübschle & Dittrich, 2011 ▸)]. In combination with the highly negatively charged nitro­gen atom, the positive value of the N1—C8 BCP Laplacian can be understood. The valence shell charge concentration (VSCC) on the nitro­gen atom is pointing to a partially positive carbon atom. The areas of VSCC in Figs. 2 ▸(*a*)–2 ▸(*f*) and 3 ▸(*a*)–3 ▸(*d*) are marked with red arrows. Such a (or related) disagreement between MM and DFT results for a certain bond was discussed in our previous paper (Vénosová *et al.*, 2020 ▸). A positive Laplacian can also be caused by a systematic error, but our MM interpretation is realistic from a chemical point of view, although hardly convincing from the DFT perspective. The experimental static deformation densities of both di­phenyl rings are shown in Figs. 4 ▸(*a*)–4 ▸(*f*). In Table 5 ▸, we see that all DFT charges are lower in absolute value compared with the experimental ones.

**Figure 5:**
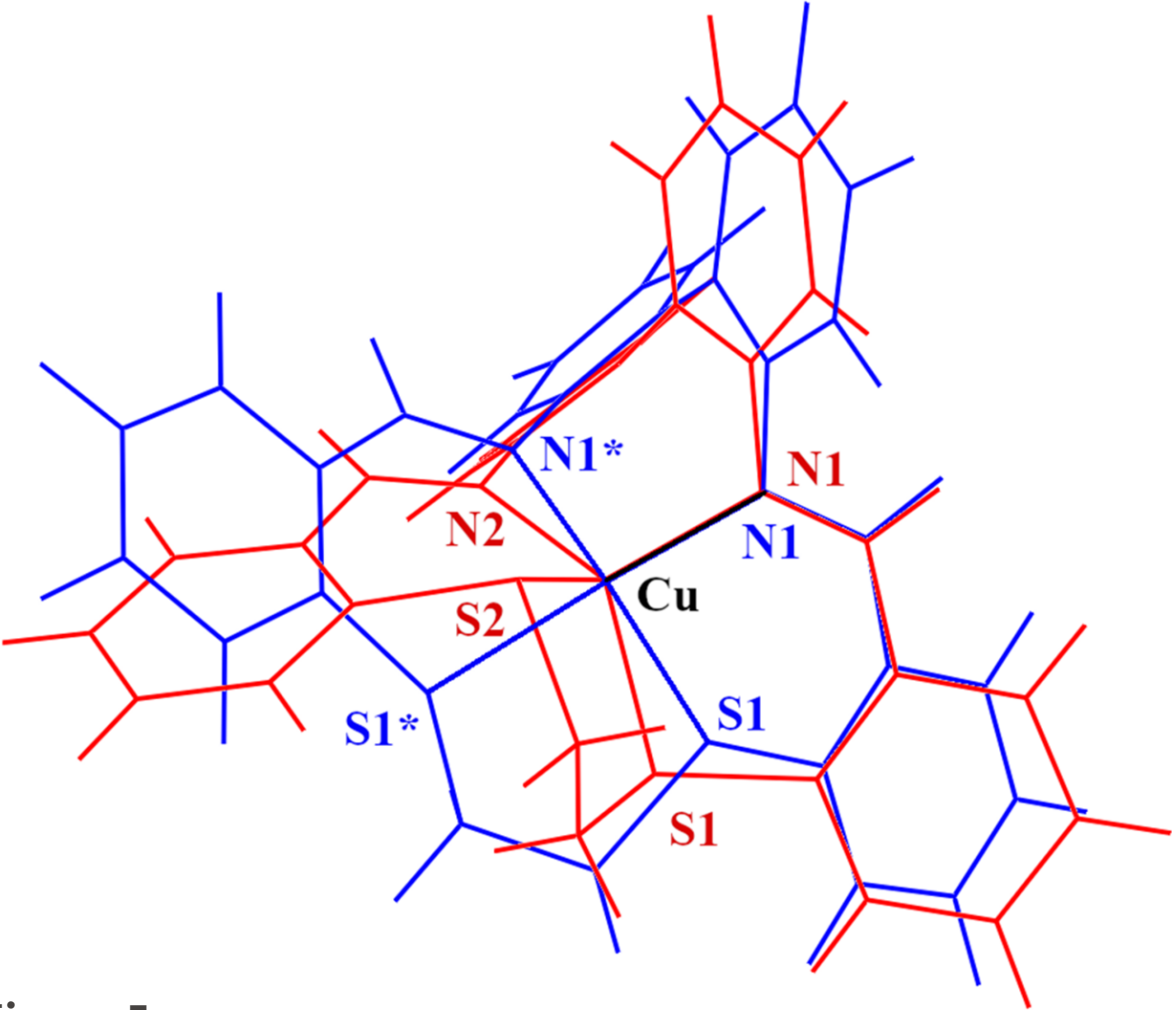
Comparison of ligands in **A** (red) and **B** (blue). The Cu—N1 bond is identical for both **A** and **B**. The symbol * indicates a symmetry-equivalent atom in **B** with symmetry code 1 − *x*, 3/2 − *y*, +*z*.

**Figure 6:**
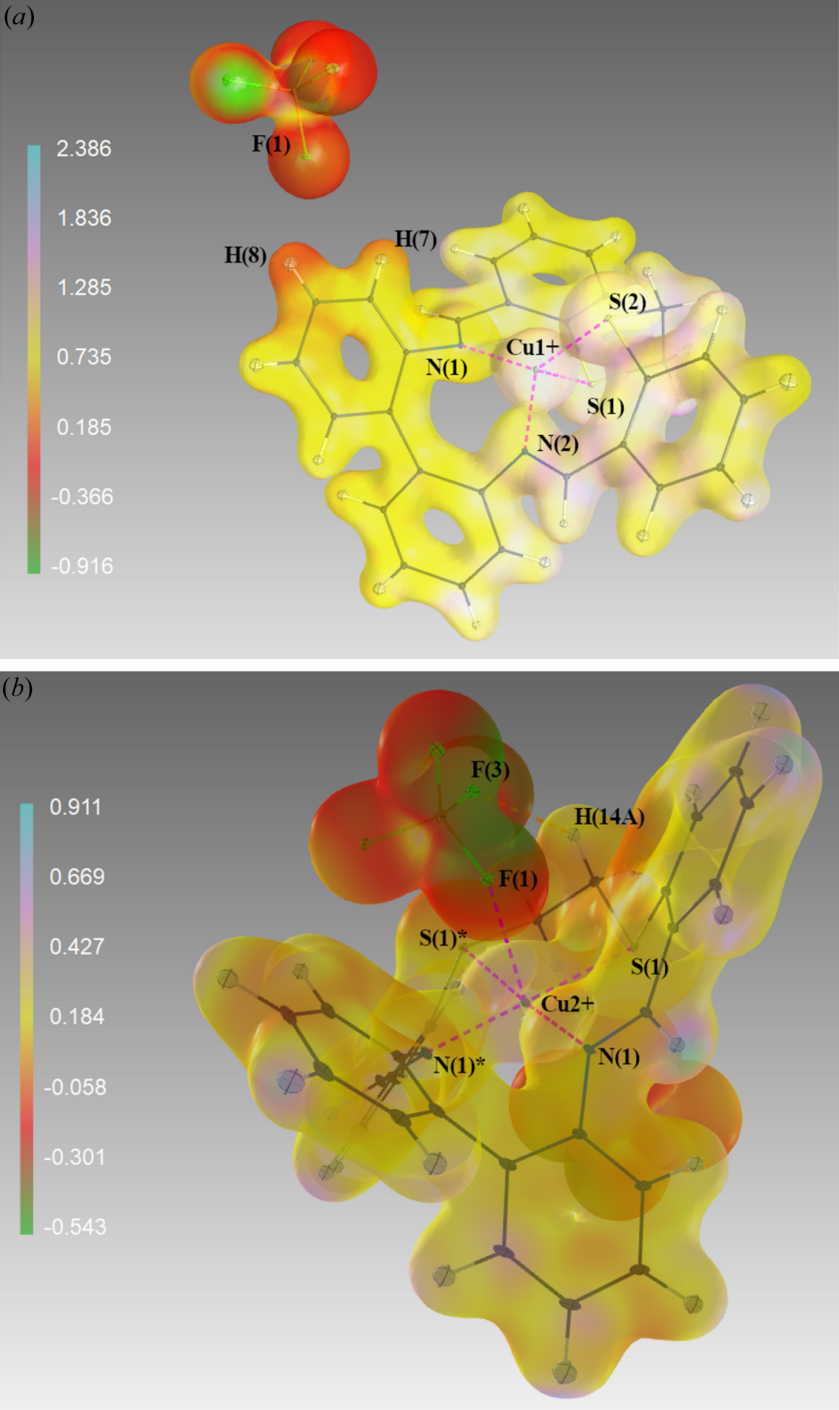
ESPs derived by *XDPROP* (Volkov *et al.*, 2016 ▸) for (*a*) **A** and the asymmetric part for (*b*) **B**. 3D plot made using *MoleCoolQt* (Hübschle & Dittrich, 2011 ▸). The isodensity surface is drawn at 0.2 e Å^−3^. The ESP values are given in the interval −0.916 to 2.386 e Å^−1^ for **A** and in the interval −0.543 to 0.911 e Å^−1^ for **B**.

This is not the case for the bi­phenyl carbon atoms, for which in **A** the above trend is valid, but in **B** the theoretical charges do not agree with the experimental results. Also, the charges on the sulfur atoms in the theoretical results do not differ between **A** and **B**. Theoretical results with optimized geometry seem unable to interpret the asymmetry in the coordination polyhedron of the central copper(I) atom in **A**. The above differences between experimental and DFT data are prevailingly ascribed to missing influences of neighboring ions in theoretical model systems. The *bite* ligand in both complexes has been adapted to the conformation required by the central atom (Fig. 5 ▸). In the case of compound **B**, the ADPs are much higher than for compound **A**. This is not an effect of absorption and secondary extinction (since one was treated and the other was found to be negligible, respectively), so the plausible explanation of the higher ADPs in **B** is the strain in the ligand due to geometrical distortions to adapt to the crystal field enforced by the central atom. The angle between the planes defined by the two benzene rings bonded to the nitro­gen atoms is 65.33° for **A** and 50.55° for **B**. In **B**, these rings are symmetrically dependent. The unusual shape of the thermal ellipsoids of these atoms is due to this compound’s symmetry, where the direction of the semi-major axis of the ellipsoids of these atoms is parallel to the intersection of these two planes. Figs. 6 ▸(*a*) and 6 ▸(*b*) show the electrostatic potentials (ESPs) of **A** and **B**. The ESP is more polarized on **A** (−0.916–2.386 e Å^−1^) and in **B** it is only −0.543–0.911 e Å^−1^. In **A**, a more positive ESP is located over the N2-benzyl­idene group together with the metallocycle S1–C5–C6–S2–Cu^+^. The ESP in **B** is more evenly distributed, consistent with the symmetry of the molecule. The Cu—N1 and Cu—N2 bonds in **A** are comparable to the Cu—N1 bond in **B**. On the contrary, the Cu—N1 bond in **B** is the average of the Cu—S1 and Cu—S2 bond values in **A**. **B** also has the Cu—F bond in the axial position identified with the AIM analysis, which is a weak bond (ρ_BCP_ and ∇^2^ρ_BCP_ have values of 0.134 and 2.643 e Å^−5^, respectively). Inspecting the 3D VSCC reveals that around the central atom there are σ-bonds (Cu—N and Cu—S). The Cu—F bond in **B** appears to be a weak coordination bond or a non-bonding interaction also according to the *d_z_*^2^ orbital population of 1.82 e and *d_yz_* orbital population of 1.71 e (see Table 6 ▸; Sabino & Coppens, 2003 ▸).

From the error analysis, it can be seen that the reflections with sin θ/λ > 0.7 Å^−1^ suffer from huge noise, and since these reflections do not provide information about the valence electron density, we did a multipole refinement for compound **B** with trimmed data (sin θ/λ < 0.7 Å^−1^). It is clear from Table S3 that the AIM analyses for both refinements are very close to each other. The results of *SHELXL* IAM refinement are given in Table S4.

## 4. Conclusions

The main results from the obtained experimental (MM-derived) and DFT electronic structures for the Cu(I) and Cu(II) systems of **A** and **B**, respectively, are to be summarized as follows:

(1) The results of the AIM analysis for experimental and DFT data in experimental geometries are consistent. Theoretical AIM atomic charges are less polarized (lower in magnitude) than multipole model AIM charges. Similar is true for the BCP electron density and Laplacian, except in the case of the N1—C8 BCP Laplacian of **B**. The question is whether the experiment can provide evidence of a new quality that the theoretical calculation does not see, *e.g.* due to crystal environment polarization or bias from systematic errors.

(2) From the AIM analysis, the strongest coordination (dative) bond in both **A** and **B** is Cu—N1, and the weakest bond in **A** is Cu—S1 and in **B** is Cu—F1. Opposite trends between DFT calculations in experimental and optimized geometries are found in **A** for the Cu—S1 and Cu—S2 bonds. Differences for DFT data in optimized geometries are caused by missing interactions with neighboring molecules.

(3) The valence shell charge concentration (VSCC) in both compounds indicates a σ character for all Cu—N and Cu—S bonds. Thus, an experimental electronic structure captures the redistribution of valence electron density in a straightforward manner.

Finally, it can be concluded that our treatment in the study of the blue protein model system is based on a suitable combination of experimental and theoretical methods of quantum crystallography. Such an approach brings valuable results and motivation for future studies on matching the experimentally derived MM electron density with the theoretical (DFT) one. In the MM, the electron density is not too biased by the actual model choice, covering all the information from the experimental data (including crystal environment and systematic errors). The theoretically determined electron density is model (DFT) choice dependent, here also lacking the crystal environment, but there are no systematic errors.

## Supplementary Material

Crystal structure: contains datablock(s) global, A_IAM, B_IAM, A_MM, B_MM. DOI: 10.1107/S2052252524012107/lq5057sup1.cif

Structure factors: contains datablock(s) A_IAM. DOI: 10.1107/S2052252524012107/lq5057A_IAMsup2.hkl

Structure factors: contains datablock(s) B_IAM. DOI: 10.1107/S2052252524012107/lq5057B_IAMsup3.hkl

Supporting figures and tables. DOI: 10.1107/S2052252524012107/lq5057sup4.pdf

CCDC references: 2327643, 2327645, 2415832, 2415833

## Figures and Tables

**Figure 3:**
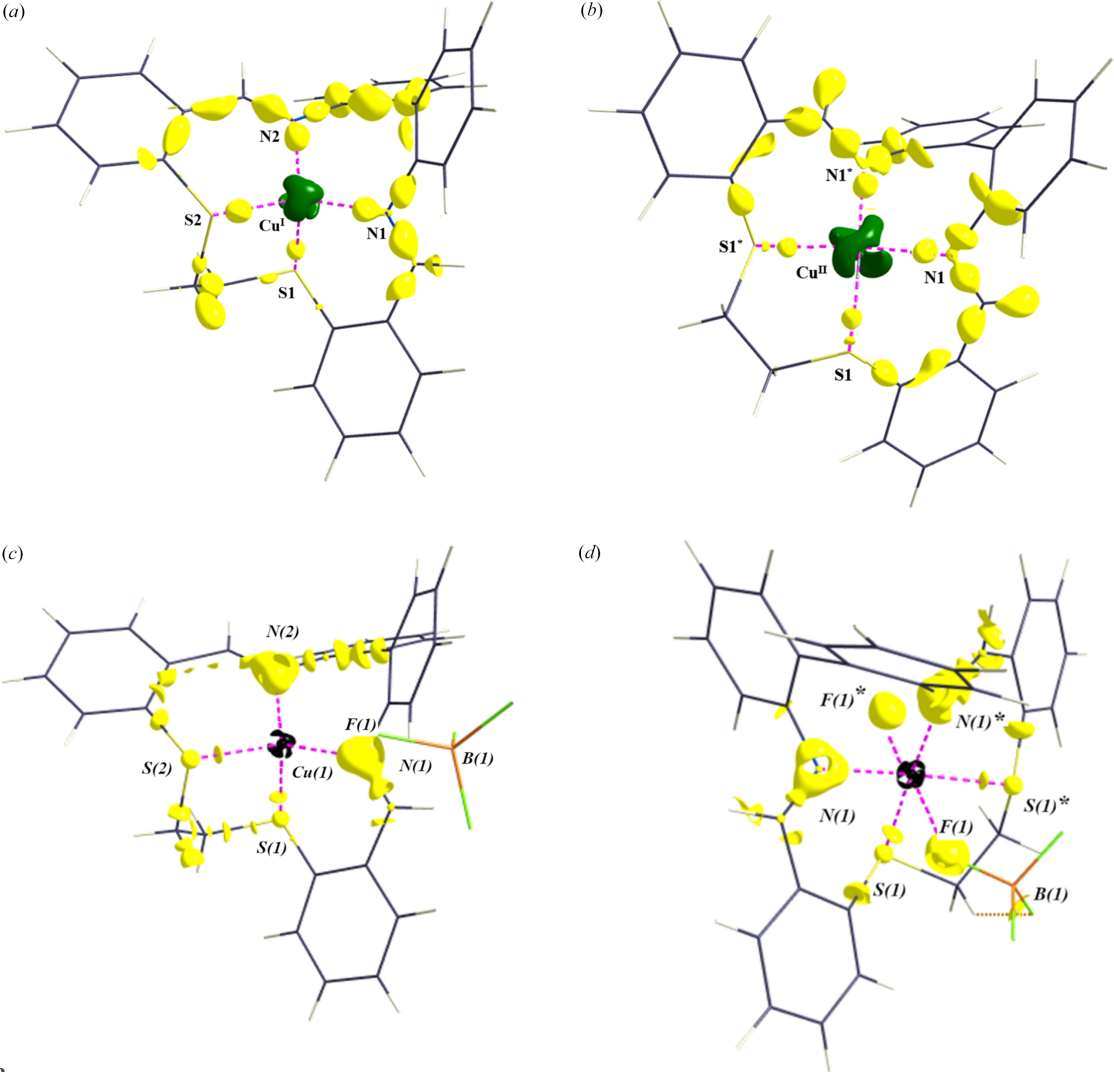
3D plots of the static deformation density and the Laplacian of the electron density in **A** and **B**. (*a*) 3D plot (Hübschle & Dittrich, 2011 ▸) of the static deformation density for compound **A** at an isosurface value of 0.3 e Å^−3^ and for the Cu atom at an isosurface value of 0.6 e Å^−3^. (*b*) 3D plot (Hübschle & Dittrich, 2011 ▸) of the static deformation density for compound **B** at an isosurface value of 0.3 e Å^−3^ and for the Cu atom at an isosurface value of 0.6 e Å^−3^. (*c*) 3D plot (Hübschle & Dittrich, 2011 ▸) of the Laplacian of the electron density for **A** at an isosurface value of 17 e Å^−5^ and around Cu at an isosurface value of 1670 e Å^−5^. (*d*) 3D plot (Hübschle & Dittrich, 2011 ▸) of the Laplacian of the electron density for **B** at an isosurface value of 17 e Å^−5^ and around Cu at an isosurface value of 1670 e Å^−5^.

**Table 1:** Experimental details

	**A**	**B**
Empirical formula	[Cu^I^(C_28_H_22_N_2_S_2_)]BF_4_	[Cu^II^(C_28_H_22_N_2_S_2_)](BF_4_)_2_
Formula weight, *Z*	600.94, 4	687.75, 8
*F*(000)	1224	2776
Temperature (K)	100.0 (1)	100.0 (1)
Crystal size (mm)	0.310 × 0.115 × 0.115	0.195 × 0.146 × 0.048
*a* (Å)	7.8890 (1)	11.6491 (4)
*b* (Å)	14.6682 (1)	11.6491 (4)
*c* (Å)	21.2891 (2)	39.773 (2)
α, β, γ (°)	90, 90, 90	90, 90, 90
*V* (Å^3^)	2463.52 (3)	5397.3 (5)
Space group	No. 19, *P*2_1_2_1_2_1_	No. 88, *I*4_1_/*a*
Wavelength (Å)	0.56083	0.56083
μ (mm^−1^)	0.582	0.548
*T*_min_, *T*_max_	0.813, 0.751	0.3626, 0.9001
Scan type	ω	ω
Max sin θ/λ (Å^−1^)	1.297	1.220
Range of indices for *h*	±11	±28
Range of indices for *k*	±20	−14, +28
Range of indices for *l*	±30	−96, +97
No. of measured reflections	144848	182450
Redundancy (all)	11.7	9.17
X-ray tube (kV), (µA)	50, 880	50, 880
Crystal-to-detector distance (mm)	40	40
*R*_int_ (for resolution 0.65 Å)	0.108	0.123
*R*(σ) (for resolution 0.65 Å)	0.150	0.280

*SHELXL* (IAM) refinement		
No. of independent reflections	7512	20608
*R*_int_, *R*_σ_	0.0326, 0.0156	0.1508, 0.2086
No. of data, restraints, parameters	7512, 0, 431	20608, 0, 235
Goof on *F*^2^	1.015	0.725
Final *R* indexes [*I* > 2σ(*I*)]	*R*_1_ = 0.0170, *wR*_2_ = 0.0465	*R*_1_ = 0.0380, *wR*_2_ = 0.0757
Final *R* indexes (all data)	*R*_1_ = 0.0181, *wR*_2_ = 0.0468	*R*_1_ = 0.2013, *wR*_2_ = 0.0949
Δρ_max_, Δρ_min_ (e Å^−3^)	0.43, −0.19	0.80, −1.37

Multipole (MM) refinement on *F*^2^		
*R*(*F*), *wR*(*F*), GooF	0.0206, 0.032, 0.7008	0.02, 0.03, 1.202
No. of reflections, parameters	155761, 987	23768, 559
Δρ_max,_ Δρ_min_ (e Å^−3^)	0.36, −0.34	0.25, −0.25

**Table 2:** Selected bond distances (Å) and angles (°) in complexes **A** and **B**

Bond in **A**:	Cu1—N1	Cu1—N2	Cu1—S1	Cu1—S2	S1—C5	S1—C17	S2—C6	S2—C24
Bond in **B**:	Cu1—N1	Cu1—N1†	Cu1—S1	Cu1—S1†	S1—C14	S1—C1	S1†—C14†	S1†—C1†
**A**	1.9406 (6)	1.9609 (5)	2.3264 (1)	2.1941 (1)	1.8242 (5)	1.7908 (5)	1.8265 (6)	1.7961 (6)
**B**	1.9855 (12)	1.9855 (12)	2.2922 (4)	2.2922 (4)	1.8303 (10)	1.7764 (10)	1.8303 (10)	1.7764 (10)

Bond in **A**:	N1—C1	N1—C15	N2—C4	N2—C22				
Bond in **B**:	N1—C8	N1—C3	N1†—C8†	N1†—C3†				
**A**	1.4276 (7)	1.2839 (7)	1.4236 (7)	1.2877 (7)				
**B**	1.4354 (16)	1.2869 (13)	1.4354 (16)	1.2869 (13)				

Angle in **A**:	S1—Cu—S2		S1—Cu—N1		S1—Cu—N2		S2—Cu—N1	
Angle in **B**:	S1—Cu—S1†		S1—Cu—N1		S1—Cu—N1†		S1†—Cu—N1	
**A**	97.758 (4)		97.44 (2)		109.81 (2)		132.99 (2)	
**B**	89.78 (2)		87.50 (4)		176.69 (4)		176.69 (4)	

Angle in **A**:	S2—Cu—N2		N1—Cu—N2		–		–	
Angle in **B**:	S1†—Cu—N1†		N1—Cu—N1†		S1—Cu—F1		N1—Cu—F1	
**A**	105.05 (2)		110.96 (2)		–		–	
**B**	87.50 (4)		95.28 (8)		91.52 (5)		93.55 (6)	

† Symmetry code: 1 − *x*, 3/2 − *y*, +*z*.

**Table 3:** Selected experimental topological properties associated with BCPs of complexes **A** and **B** *R_ij_* – bond path length, *d*_1_ – first atom-to-BCP distance, ρ_c_ – BCP electron density, ∇^2^ρ_c_ – BCP Laplacian.

	*R_ij_* (Å)		*d*_1_ (Å)		ρ_c_ (e Å^−3^)		∇^2^ρ_c_ (e Å^−5^)		Ellipticity	
Bond	**A**	**B**	**A**	**B**	**A**	**B**	**A**	**B**	**A**	**B**
Cu1—N1†	1.9407	1.9855	0.9750	0.9854	0.691 (5)	0.671 (6)	11.257 (7)	9.943 (8)	0.10	0.05
Cu1—N1‡	1.9405	1.9868	0.9492	0.9593	0.679	0.634	10.685	8.150	0.060	0.046
Cu1—N1§	1.9723	2.0007	0.9628	0.9649	0.622	0.617	9.593	7.803	0.033	0.043
Cu1—N2†	1.9612	–	0.9797	–	0.671 (4)	–	10.603 (6)	–	0.12	–
Cu1—N2‡	1.9620	1.9868	0.9602	0.9593	0.645	0.634	10.018	8.150	0.024	0.046
Cu1—N2§	1.9603	2.0007	0.9567	0.9649	0.639	0.617	9.890	7.803	0.054	0.043
Cu1—S1†	2.3268	2.2927	1.0754	1.0535	0.442 (3)	0.511 (4)	5.329 (3)	6.075 (4)	0.02	0.07
Cu1—S1‡	2.3264	2.2919	1.0625	1.0360	0.459	0.515	4.465	3.861	0.012	0.015
Cu1—S1§	2.2495	2.3280	1.0295	1.0494	0.526	0.482	5.135	3.533	0.026	0.013
Cu1—S2†	2.1942	–	1.0210	–	0.598 (4)	–	7.024 (4)	–	0.05	–
Cu1—S2‡	2.1944	2.2919	1.0091	1.0360	0.596	0.515	5.940	3.861	0.036	0.015
Cu1—S2§	2.3347	2.3282	1.0645	1.0494	0.445	0.481	4.265	3.530	0.014	0.013
Cu1—F1†	–	2.5302	–	1.2900	–	0.134 (1)	–	2.643 (3)	–	0.06
Cu1—F1‡	–	–	–	–	–	–	–	–	–	–
Cu1—F1§	–	–	–	–	–	–	–	–	–	–

† Experimental.

‡ DFT in X-ray geometries.

§ DFT in optimized geometries.

**Table 4:** The strongest experimental non-bonding interactions Topological properties associated with BCPs of complexes [Cu(*bite*)]^+^, **A**, and [Cu(*bite*)]^2+^, **B**. *R_ij_* – bond path length, *d*_1_ – first atom-to-BCP distance, ρ_c_ – BCP electron density, ∇^2^ρ_c_ – BCP Laplacian, ellipticity, *E*_TOT_, calculated following the paper by Espinosa *et al.*, 1998 ▸.

Bond type	*R_ij_* (Å)	*d*_1_ (Å)	ρ_c_ (e Å^−3^)	∇^2^ρ_c_ (e Å^−5^)	Ellipticity	*E*_TOT_ (kJ mol^−1^)
*D*—H⋯*A* for **A**						
C(8)—H(8)⋯F(1)	2.3949	1.3496	0.076 (1)	1.257 (1)	0.04	77.0
C(25)†—H(25)†⋯F(4)	2.4894	1.4602	0.039 (2)	0.691 (1)	0.36	42.3
C(9)‡—H(9)‡⋯F(3)	2.5058	1.5104	0.024 (1)	0.685 (1)	0.08	42.0

*D*—H⋯*A* for **B**						
C(14)—H(14A)⋯F(3)	2.1974	1.3149	0.093 (3)	1.621 (2)	0.06	14.7
C(3)§—H(3)§⋯F(4)	2.2497	1.3996	0.051 (4)	1.351 (1)	0.27	12.3
C(6)¶—H(6)¶⋯F(4)	2.3799	1.4082	0.055 (4)	1.011 (1)	0.07	9.2

† Symmetry code: 5/2 − *x*, 1 − *y*, −1/2 + *z*.

‡ Symmetry code: 5/2 − *x*, −1 − *y*, 1/2 + *z*.

§ Symmetry code: 3/4− *y*, 3/4 + *x*, 3/4 − *z*.

¶ Symmetry code: −*x*, 3/2 − *y*, *z*.

**Table 5:** Charges (e) on the non-hydrogen atoms for complexes **A** and **B**

Atom in **A**:	Cu1	N1	N2	S1	S2	C1	C2	C3	C4	C5	C6	C7	C8
Atom in **B**:	Cu1	N1	N1†	S1	S1†	C8	C13	C13†	C8†	C14	C14†	C9	C10
**A** experiment	0.73	−0.80	−0.99	0.21	0.25	0.12	0.11	−0.08	0.29	0.58	0.32	0.35	0.39
**A** DFT[Table-fn tfn10]	0.56	−1.24	−1.23	0.07	0.08	0.33	0.01	0.00	0.34	−0.01	−0.01	0.01	0.02
**A** DFT[Table-fn tfn11]	0.55	−1.27	−1.28	0.06	0.07	0.35	0.01	0.01	0.35	−0.00	−0.00	0.01	0.03
**B** experiment	1.22	−1.50	−1.50	0.06	0.06	−0.03	0.27	0.27	−0.03	0.13	0.13	0.08	−0.08
**B** DFT[Table-fn tfn10]	0.84	−1.25	−1.25	0.08	0.08	0.29	0.00	0.00	0.29	−0.02	−0.02	0.00	0.03
**B** DFT[Table-fn tfn11]	0.83	−1.27	−1.27	0.07	0.07	0.30	0.01	0.01	0.30	−0.02	−0.02	0.01	0.03

Atom in **A**:	C9	C10	C11	C12	C13	C14	C15	C16	C17	C18	C19	C20	C21
Atom in **B**:	C11	C12	C12†	C11†	C10†	C9†	C3	C2	C1	C4	C5	C6	C7
**A** experiment	0.18	−0.05	0.06	0.44	−0.02	0.32	0.65	0.27	0.05	0.23	0.29	0.39	0.16
**A** DFT[Table-fn tfn10]	0.02	0.01	0.01	0.02	0.02	0.00	0.74	0.01	−0.13	0.02	0.03	0.02	0.01
**A** DFT[Table-fn tfn11]	0.02	0.01	0.01	0.02	0.03	0.01	0.76	0.01	−0.13	0.02	0.03	0.03	0.02
**B** experiment	0.46	−0.16	−0.16	0.46	−0.08	0.08	0.80	−0.01	−0.05	0.18	0.07	0.19	0.0
**B** DFT[Table-fn tfn10]	0.03	0.01	0.01	0.03	0.03	0.00	0.72	0.01	−0.13	0.03	0.04	0.04	0.03
**B** DFT[Table-fn tfn11]	0.03	0.01	0.01	0.03	0.03	0.01	0.73	0.02	−0.12	0.03	0.04	0.04	0.03

Atom in **A**:	C22	C23	C24	C25	C26	C27	C28	B1	F1	F2	F3	F4	
Atom in **B**:	C3†	C2†	C1†	C4†	C5†	C6†	C7†	B1	F1	F2	F3	F4	
**A** experiment	0.69	0.19	0.02	0.17	0.35	0.48	0.06	2.31	−1.01	−0.96	−0.75	−0.58	
**A** DFT[Table-fn tfn10]	0.73	0.01	−0.13	0.02	0.02	0.02	0.01						
**A** DFT[Table-fn tfn11]	0.76	0.01	−0.13	0.02	0.03	0.03	0.02						
**B** experiment	0.80	−0.01	−0.05	0.18	0.07	0.19	0.0	2.68	−0.95	−0.84	−0.91	−0.97	
**B** DFT[Table-fn tfn10]	0.72	0.01	−0.13	0.03	0.04	0.04	0.03						
**B** DFT[Table-fn tfn11]	0.73	0.02	−0.12	0.03	0.04	0.04	0.03						

† Symmetry code: 1 − *x*, 3/2 − *y*, +*z*.

‡ Experimental geometry.

§ Optimized geometry.

**Table 6:** Population of 3*d* orbitals at Cu

Compound	*d*_*z*^2^_ (%)	*d_xz_* (%)	*d_yz_* (%)	*d*_*x*^2^ − *y*^2^_ (%)	*d_xy_* (%)	Σ
**A** (from *XDPROP*)	2.11 (3)	2.08 (3)	2.11 (3)	1.90 (3)	1.85 (3)	10.05
	21.0	20.7	21.0	18.9	18.4	
**B** (from *XDPROP*)	2.03 (3)	1.60 (3)	1.43 (3)	2.22 (3)	2.24 (3)	9.52
	21.3	16.9	15.0	23.3	24.5	
**A** (after transformation)†	2.07	1.95	1.97	1.99	2.07	10.05
	20.6	19.4	19.6	19.8	20.6	
**B** (after transformation)†	1.82	2.00	1.71	1.99	2.00	9.52
	19.1	21.0	17.9	21.0	21.0	

† Minimizing the *d*-orbital cross-terms using *jnk2RDA* as proposed by Sabino & Coppens (2003 ▸).
