# Supplementary material for: Experimental electronic structures of copper complexes with a bi­phenyldi­imino di­thio­ether – a model for blue copper proteins
Source: IUCrJ. 2025 Jan 30;12(Pt 2):198–207. doi: 10.1107/S2052252524012107 (PMC11878452; doi:10.1107/S2052252524012107)
Supplement: Supplementary file 4 [file m-12-00198-sup4.pdf]

IUCrJ

Volume 12 (2025)

Supporting information for article:

Experimental electronic structure of copper complexes with bi-phenyldiimino dithioether – model of blue copper proteins

Marek Fronc, Martin Breza, Lukáš Bučinský, Ingrid Jelemenská and Jozef Kožíšek

CONTENTS

1.	Figure 1a, 1b	ORTEP plot	2
2.	Figure 2a, 2b	Residual map	2
3.	Figure 3a-d	Variation of scale factor	3
4.	Figure 4a-c	Fractal plot of the residual density	3
5.	Table SI1	Bond distances [Å] in complexes (**A**) and (**B**)	4
6.	Table SI2a-c	Topological properties of complexes (**A**) and (**B**)	5
7.	Table SI3	Comparison of SHELXL and XD2016 refinement (**B**)	10
8.	Table SI4	Selected experimental topological properties	11

Figure S1 ORTEP plot of the compound. Thermal ellipsoids are drawn at 50% probability. ORTEP plot of the compound. Thermal ellipsoids are drawn at 50% probability.

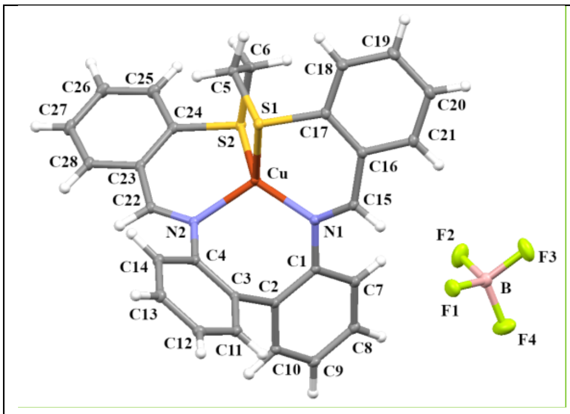

Figure S1a. ORTEP plot of the compound (**A**). Thermal ellipsoids are drawn at 50% probability.

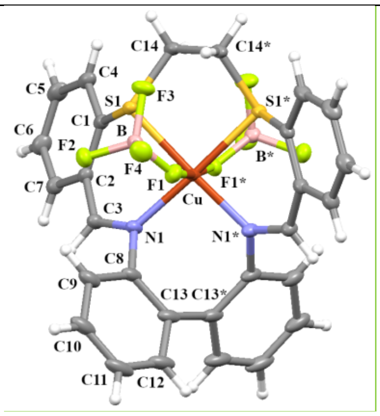

Figure S1b. ORTEP plot of the compound (**B**). Thermal ellipsoids are drawn at 50% probability. Symmetry code: *) 1 – x, 3/2 – y, z

Figure S2 Residual maps for (**A**) and (**B**). Contours are defined as in **Figures 5.**

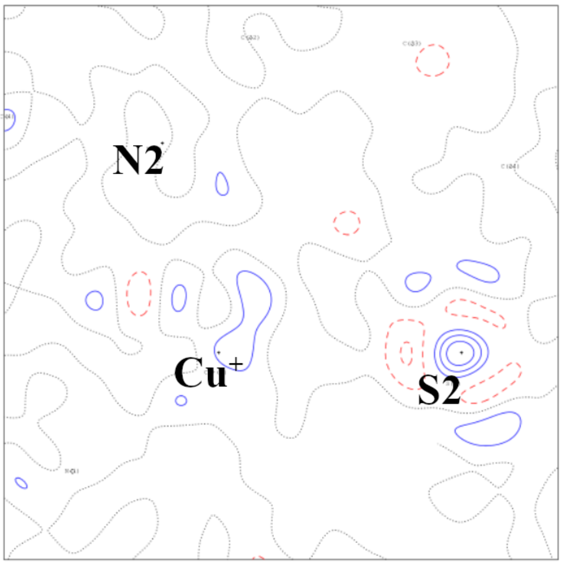

Figure S2a Residual map for (**A**). Plane defined by Cu(1), S(2), and N(2) atoms.

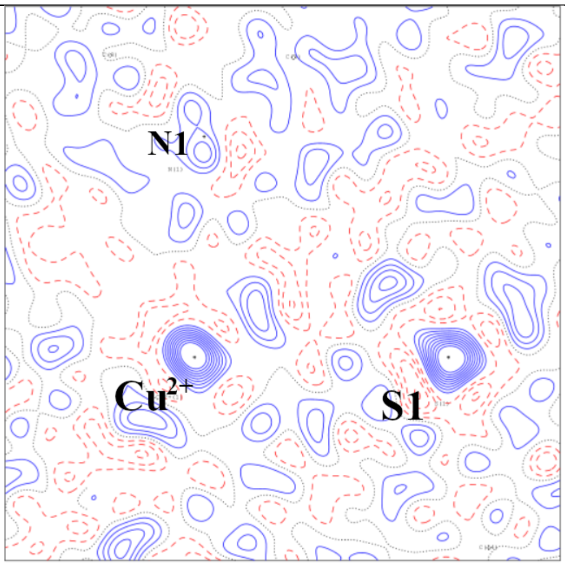

Figure S2b Residual map for (**B**). Plane defined by Cu(1), S(1), and N(1) atoms.

Figure S3 for SHELXL and XD2016 refinement.

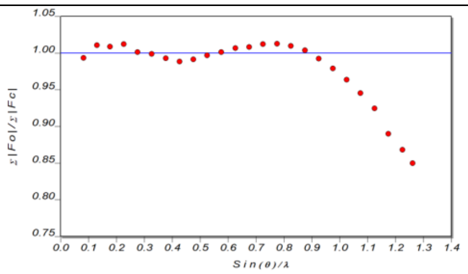

Figure S3a. Variation of scale factor[1] for (**A**) for SHELXL refinement.

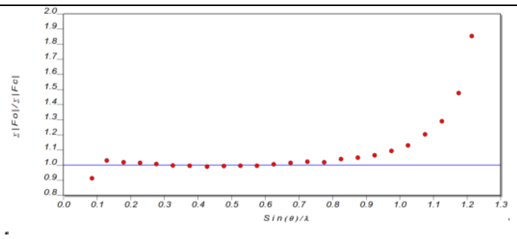

Figure S3c. Variation of scale factor[1] for (**B**) for SHELXL refinement.

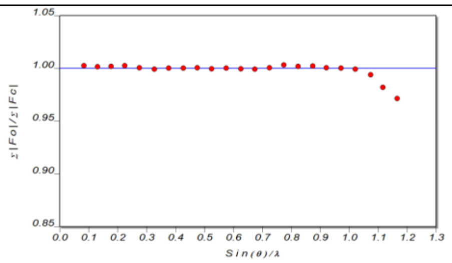

Figure S3b. Variation of scale factor[1] for (**A**) for XD2016 multipole refinement.

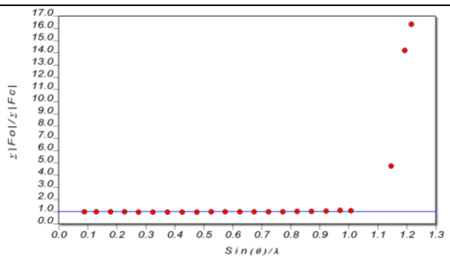

Figure S3b. Variation of scale factor[1] for (**B**) for XD2016 multipole refinement.

[1](Abrahams & Keve, 1971)

Figure S4 Fractal plot[2] of the residual density

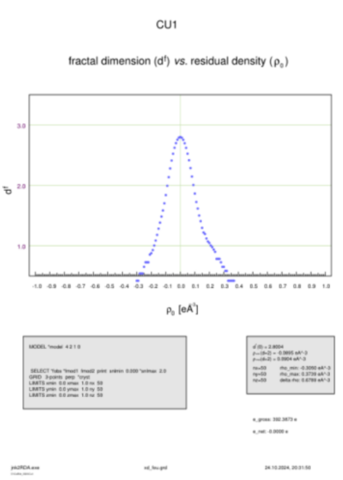

Figure S4a

Fractal plot of the residual density for (**A**)

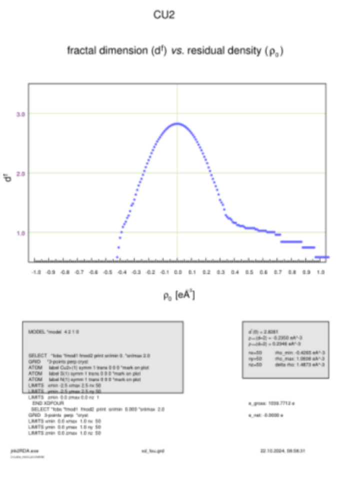

Figure S4b

Fractal plot of the residual density for (**B**)

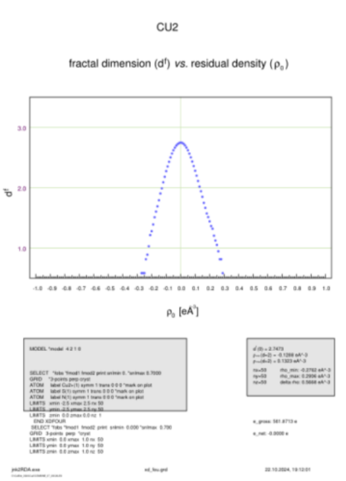

Figure S4c

Fractal plot of the residual density for (**B**)*

[2](Meindl & Henn, 2008). * data is trimmed at d_{min} = 0.70 Å

Table S1 Bond distances [Å] in complexes (**A**) and (**B**)

**Complex/ bond**	**Cu1-N1**<br>**Cu1-N1**	**Cu1-N2**<br>**Cu1-N1***	**Cu1-S1**<br>**Cu1-S1**	**Cu1-S2**<br>**Cu1-S1***	**S1-C5**<br>**S1-C14**	**S1-C17**<br>**S1-C1**	**S2-C6**<br>**S1*-C14***	**S2-C24**<br>**S1*-C1***
(**A**)	1.9406(6)	1.9609(5)	2.3264(1)	2.1941(1)	1.8242(5)	1.7908(5)	1.8265(6)	1.7961(6)
(**B**)	1.9855(12)	1.9855(12)	2.2922(4)	2.2922(4)	1.8303(10)	1.7764(10)	1.8303(10)	1.7764(10)
**Complex/ bond**	**N1-C1**<br>**N1-C8**	**N1-C15**<br>**N1-C3**	**N2-C4**<br>**N1*-C8***	**N2-C22**<br>**N1*-C3***	**C1-C2**<br>**C8-C13**	**C1-C7**<br>**C8-C9**	**C2-C3**<br>**C13-C13***	**C2-C10**<br>**C13-C12**
(**A**)	1.4276(7)	1.2839(7)	1.4236(7)	1.2877(7)	1.4093(7)	1.3971(6)	1.4992(7)	1.4015(7)
(**B**)	1.4354(16)	1.2869(13)	1.4354(16)	1.2869(13)	1.4080(17)	1.3978(16)	1.481(3)	1.4000(18)
**Complex/ bond**	**C3-C4**<br>**C13*-C8***	**C3-C11**<br>**C13*-C12***	**C4-C14**<br>**C8*-C9***	**C5-C6**<br>**C14-C14***	**C7-C8**<br>**C9-C10**	**C8-C9**<br>**C10-C11**	**C9-C10**<br>**C11-C12**	**C11-C12**<br>**C12*-C11***
(**A**)	1.4169(7)	1.4043(6)	1.4058(6)	1.5240(7)	1.3968(7)	1.3967(7)	1.3975(7)	1.3988(7)
(**B**)	1.4080(17)	1.4000(18)	1.3978(16)	1.5338(18)	1.3878(17)	1.400(2)	1.389(2)	1.389(2)
**Complex/ bond**	**C12-C13**<br>**C11*-C10***	**C13-C14**<br>**C10*-C9***	**C15-C16**<br>**C3-C2**	**C16-C17**<br>**C2-C1**	**C16-C21**<br>**C2-C7**	**C17-C18**<br>**C1-C4**	**C18-C19**<br>**C4-C5**	**C19-C20**<br>**C5-C6**
(**A**)	1.3931(8)	1.3957(7)	1.4736(7)	1.4198(7)	1.4101(7)	1.3996(7)	1.3951(7)	1.3942(8)
(**B**)	1.3967(7)	1.3878(17)	1.4694(14)	1.4056(17)	1.4067(12)	1.3989(14)	1.4004(15)	1.390(2)
**Complex/ bond**	**C20-C21**<br>**C6-C7**	**C22-C23**<br>**C3*-C2***	**C23-C24**<br>**C2*-C1***	**C23-C28**<br>**C2*-C7***	**C24-C25**<br>**C1*-C4***	**C25-C26**<br>**C4*-C5***	**C26-C27**<br>**C5*-C6***	**C27-C28**<br>**C6*-C7***
(**A**)	1.3956(7)	1.4716(7)	1.4216(7)	1.4085(7)	1.3982(7)	1.3968(8)	1.3960(8)	1.3911(8)
(**B**)	1.3894(16)	1.4694(14)	1.4056(17)	1.4067(12)	1.3989(14)	1.4004(15)	1.390(2)	1.3894(16)
**Complex/ bond**	**B1-F1**	**B1-F2**	**B1-F3**	**B1-F4**	**Cu1-F1**			
(**A**)	1.4033(9)	1.3931(11)	1.4196(12)	1.4060(13)	-			
(**B**)	1.4178(19)	1.4018(17)	1.406(2)	1.3927(17)	2.5301(19)			

Symmetry code: *) 1 - X, 3/2 - Y, + Z

Table S2a Topological properties associated with bond critical points of complexes (A) and (B) for the multipolar model in X-ray geometry (R_{ij} – the bond path length, d_1 – the first atom-to-BCP distance, ρ_c – BCP electron density, $\nabla^2\rho_c$ – BCP Laplacian).

	**R_{ij} [Å]**		**d_1 [Å]**		**ρ_c [e Å^{-3}]**		**$\nabla^2\rho_c$ [e Å^{-5}]**		**ellipticity**	
**Bond type / Complex**	**(A)**	**(B)**	**(A)**	**(B)**	**(A)**	**(B)**	**(A)**	**(B)**	**(A)**	**(B)**
Cu1-N1	1.9407	1.9855	0.9750	0.9854	0.691(5)	0.671(6)	11.257(7)	9.943(8)	0.10	0.05
Cu1-N2	1.9612	-	0.9797	-	0.671(4)	-	10.603(6)	-	0.12	-
Cu1-S1	2.3268	2.2927	1.0754	1.0535	0.442(3)	0.511(4)	5.329(3)	6.075(4)	0.02	0.07
Cu1-S2	2.1942	-	1.0210	-	0.598(4)	**-**	7.024(4)	-	0.05	--
Cu1-F1	-	2.5302	-	1.2900	-	0.134(1)	-	2.643(3)	-	0.06
S1-C5 / S1-C14	1.8257	1.8305	0.9784	0.9475	1.20(1)	1.15(1)	-3.76(2)	-3.60(3)	0.02	0.08
S1-C17 / S1-C1	1.7910	1.7771	0.9404	0.8989	1.27(1)	1.35(1)	-4.06(2)	-6.08(3)	0.23	0.23
S2-C6	1.8267	-	0.9383	-	1.20(1)	-	-3.01(2)	-	0.13	-
S2-C24	1.7977	-	0.9210	-	1.19(1)	-	-3.31(3)	-	0.12	-
N1-C1 / N1-C8	1.4287	1.4433	0.8036	1.0026	1.95(2)	1.38(6)	-11.70(6)	7.5(3)	0.14	0.33
N1-C15 / N1-C3	1.2843	1.2869	0.6761	0.8612	2.812)	2.49(5)	-27.19(8)	-17.0(3)	0.24	0.24
N2-C4	1.4241	-	0.8191	-	1.97(2)	-	-17.15(6)	-	0.09	-
N2-C22 CH	1.2882	-	0.8200	-	2.55(3)	-	-32.78(12)	-	0.25	-
C1-C2 / C8-C13	1.4096	1.4111	0.7459	0.8836	2.09(2)	1.88(7)	-18.31(5)	-12.6(3)	0.21	0.32
C1-C7 / C8-C9	1.3971	1.3992	0.7207	0.7690	2.11(2)	2.19(4)	-18.05(4)	-19.4(1)	0.28	0.20
C2-C3 / C13-C13	1.4993	1.4815	0.7530	0.7407	1.75(1)	1.86(5)	-11.78(3)	-11.70(6)	0.05	0.10
C2-C10 / C13-C12	1.4018	1.4011	0.6542	0.7030	2.05(2)	1.90(3)	-16.32(5)	-13.93(8)	0.27	0.33
C3-C4	1.4172	-	0.7045	-	2.13(2)	-	-17.86(4)	-	0.24	-
C3-C11 / C13-C12	1.4044	-	0.7060	-	2.07(2)	-	-16.55(4)	-	0.31	-
C4-C14	1.4059	-	0.7230	-	2.20(2)	-	-19.93(4)	-	0.24	-

C5-C6 / C14-C14	1.5250	1.5355	0.7981	0.7743	1.72(1)	1.66(2)	-11.70(3)	-10.22(2)	0.01	0.14
C7-C8 / C9-C10	1.3974	1.3888	0.7165	0.6651	2.13(2)	1.97(4)	-18.26(5)	-15.13(8)	0.17	0.31
C8-C9 / C10-C11	1.3968	1.4007	0.6663	0.6984	2.11(2)	1.92(4)	-17.17(5)	-11.52(9)	0.22	0.13
C9-C10 / C11-C12	1.3975	1.3922	0.6829	0.6193	2.14(2)	2.11(5)	-17.62(4)	-15.9(1)	0.25	0.16
C11-C12 / C12-C11	1.3992	-	0.7516	-	2.18(2)	-	-18.50(5)	-	0.22	-
C12-C13 / C11-C10	1.3932	-	0.6867	-	2.17(2)	-	-19.76(5)	-	0.15	-
C13-C14 / C10-C9	1.3958	-	0.7169	-	2.19(2)	-	-20.78(4)	-	0.21	-
C15-C16 / C3-C2	1.4737	1.4699	0.7543	0.6575	1.91(2)	1.81(3)	-15.50(4)	-13.53(7)	0.22	0.10
C16-C17 / C2-C1	1.4201	1.4058	0.6916	0.6833	2.05(2)	1.89(3)	-16.91(4)	-12.96(8)	0.21	0.42
C16-C21 / C2-C7	1.4101	1.4075	0.6350	0.7513	2.14(2)	2.17(3)	-20.20(5)	-18.59(8)	0.16	0.21
C17-C18 / C1-C4	1.3997	1.3990	0.6414	0.6257	2.06(2)	2.01(3)	-16.70(5)	-16.60(9)	0.27	0.12
C18-C19 / C4-C5	1.3952	1.4015	0.7014	0.6240	2.14(2)	2.17(3)	-18.60(5)	-20.13(9)	0.18	0.22
C19-C20 / C5-C6	1.3947	1.3898	0.6743	0.7063	2.17(2)	1.92(4)	-18.25(5)	-14.45(8)	0.18	0.31
C20-C21 / C6-C7	1.3956	1.3895	0.6849	0.6180	2.17(2)	2.03(4)	-19.46(4)	-17.6(1)	0.17	0.17
C22-C23	1.4723	-	0.7700	-	1.89(1)	-	-15.19(4)	-	0.13	-
C23-C24	1.4232	-	0.7146	-	1.94(2)	-	-15.09(4)	-	0.34	-
C23-C28	1.4085	-	0.6553	-	2.12(2)	-	-19.40(5)	-	0.14	-
C24-C25	1.3984	-	0.6692		2.19(2)	-	-20.69(5)	-	0.32	-
C25-C26	1.3968	-	0.6980	--	2.23(2)	-	-21.46(4)	-	0.20	-
C26-C27	1.3962	-	0.7080	-	2.17(2)	-	-18.98(5)	-	0.28	-
C27-C28	1.3912	-	0.6750	-	2.22(2)	-	-21.53(5)	-	0.14	-
B1-F1	1.4037	1.4248	0.9328	0.9645	1.19(1)	0.84(2)	13.54(6)	29.67(8)	0.13	0.52
B1-F2	1.3936	1.4063	0.9240	0.9543	1.13(1)	1.08(2)	20.27(6)	24.59(8)	0.47	0.14
B1-F3	1.4230	1.4205	0.9352	0.9602	1.03(1)	0.99(2)	10.90(7)	24.07(9)	0.15	0.08
B1-F4	1.4436	1.3970	0.9319	0.9425	1.00(1)	1.09(2)	4.95(12)	25.21(8)	0.62	0.16

Table S2b Topological properties associated with bond critical points of complexes $[Cu(bite)]^{+}$ (A) and $[Cu(bite)]^{2+}$ (B) for DFT in X-ray geometries (R_{ij} – the bond path length, d_1 – the first atom-to-BCP distance, ρ_c – BCP electron density, $\nabla^2\rho_c$ – BCP Laplacian).

	R_{ij} [Å]		d_1 [Å]		ρ_c [e Å^{-3}]		$\nabla^2\rho_c$ [e Å^{-5}]		ellipticity	
**Bond type / Complex**	**(A)**	**(B)**	**(A)**	**(B)**	**(A)**	**(B)**	**(A)**	**(B)**	**(A)**	**(B)**
Cu1-N1	1.9405	1.9868	0.9492	0.9593	0.679	0.634	10.685	8.150	0.060	0.046
Cu1-N2	1.9620	1.9868	0.9602	0.9593	0.645	0.634	10.018	8.150	0.024	0.046
Cu1-S1	2.3264	2.2919	1.0625	1.0360	0.459	0.515	4.465	3.861	0.012	0.015
Cu1-S2	2.1944	2.2919	1.0091	1.0360	0.596	0.515	5.940	3.861	0.036	0.015
N1-C1 / N1-C8	1.4265	1.4348	0.8637	0.8779	1.904	1.846	-18.915	-17.731	0.045	0.043
N1-C15 / N1-C3	1.2834	1.2884	0.8173	0.8185	2.520	2.499	-20.416	-21.091	0.227	0.187
C15-C16 / C3-C2	1.4751	1.4683	0.7515	0.7099	1.867	1.894	-19.242	-19.826	0.106	0.104
C16-C17 / C2-C1	1.4194	1.4072	0.7023	0.7186	2.016	2.064	-21.283	-22.373	0.195	0.201
C17-S1 / C1-S1	1.7903	1.7770	0.9278	0.9423	1.257	1.287	-6.603	-7.058	0.134	0.143
C1-C2 / C8-C13	1.4098	1.4077	0.7208	0.7244	2.079	2.080	-22.838	-22.758	0.232	0.240
C2-C3 / C13-C13	1.4987	1.4845	0.7483	0.7423	1.764	1.813	-16.951	-18.009	0.032	0.054
C3-C4 / C13-C8	1.4173	1.4077	0.6955	0.7244	2.054	2.080	-22.281	-22.758	0.228	0.240
C4-N2 / C8-N1	1.4242	1.4348	0.8613	0.8779	1.913	1.846	-18.922	-17.731	0.056	0.043
C1-C7 / C8-C9	1.3964	1.3989	0.7217	0.7258	2.128	2.114	-23.985	-23.631	0.231	0.233
C7-C8 / C9-C10	1.3954	1.3890	0.7052	0.7092	2.130	2.162	-24.146	-24.917	0.195	0.189
C8-C9 / C10-C11	1.3946	1.3993	0.7009	0.7019	2.138	2.116	-24.387	-23.951	0.193	0.184
C9-C10 / C11-C12	1.3959	1.3911	0.6935	0.6866	2.133	2.154	-24.291	-24.845	0.191	0.186
C10-C2 / C12-C13	1.4012	1.4003	0.6919	0.6835	2.102	2.110	-23.228	-23.486	0.210	0.197
C3-C11 / C13-C12	1.4033	1.4003	0.7115	0.6835	2.097	2.110	-23.072	-23.486	0.208	0.197
C11-C12 / C12-C11	1.3982	1.3911	0.7037	0.6866	2.122	2.154	-24.047	-24.845	0.191	0.186
C12-C13 / C11-C10	1.3916	1.3993	0.6926	0.6973	2.151	2.116	-24.725	-23.951	0.192	0.184

C13-C14 / C10-C9	1.3946	1.3890	0.6872	0.7092	2.130	2.162	-24.146	-24.917	0.198	0.189
C14-C4 / C9-C8	1.4053	1.3989	0.7237	0.7258	2.096	2.114	-23.156	-23.631	0.224	0.233
C17-C18 / C1-C4	1.3980	1.3974	0.7167	0.7311	2.115	2.101	-23.462	-23.117	0.205	0.211
C18-C19 / C4-C5	1.3946	1.4001	0.7027	0.7120	2.137	2.110	-24.363	-23.773	0.187	0.176
C19-C20 / C5-C6	1.3929	1.3908	0.7024	0.7042	2.151	2.163	-24.797	-25.183	0.183	0.174
C20-C21 / C6-C7	1.3930	1.3896	0.6871	0.6815	2.146	2.168	-24.580	-25.207	0.192	0.180
C21-C16 / C7-C2	1.4087	1.4071	0.7108	0.7168	2.079	2.084	-22.773	-22.965	0.188	0.184
N2-C22 CH	1.2868	1.2884	0.8176	0.8185	2.512	2.499	-20.980	-21.091	0.220	0.187
C22-C23	1.4722	1.4683	0.7478	0.7099	1.874	1.894	-19.324	-19.826	0.110	0.104
C23-C24	1.4207	1.4072	0.7057	0.7186	2.014	2.064	-21.298	-22.373	0.191	0.201
C24-S2	1.7959	1.7770	0.9320	0.9423	1.247	1.287	-6.463	-7.058	0.079	0.143
C24-C25	1.3978	1.3974	0.7129	0.7311	2.122	2.101	-23.611	-23.117	0.206	0.211
C25-C26	1.3963	1.4001	0.7026	0.7120	2.134	2.110	-24.339	-23.773	0.182	0.176
C26-C27	1.3940	1.3908	0.7008	0.7042	2.148	2.163	-24.725	-25.183	0.181	0.174
C27-C28	1.3901	1.3896	0.6868	0.6815	2.162	2.168	-24.990	-25.207	0.191	0.180
C28-C23	1.4083	1.4071	0.7100	0.7168	2.087	2.084	-23.011	-22.965	0.182	0.184
S1-C5 / S1-C14	1.8234	1.8294	0.9647	0.9837	1.175	1.170	-5.299	-5.338	0.085	0.077
S2-C6	1.8260	1.8294	0.9688	0.9837	1.169	1.170	-5.169	-5.338	0.076	0.077
C5-C6 / C14-C14	1.5229	1.5357	0.7607	0.7679	1.672	1.616	-14.654	-13.382	0.012	0.004

Table S2c Topological properties associated with bond critical points of complexes $[Cu(bite)]^{+}$ (A) and $[Cu(bite)]^{2+}$ (B) in DFT optimized geometries (R_{ij} – the bond path length, d_1 – the first atom-to-BCP distance, ρ_c – BCP electron density, $\nabla^2\rho_c$ – BCP Laplacian).

	**R_{ij} [Å]**		**d_1 [Å]**		**ρ_c [e Å^{-3}]**		**$\nabla^2\rho_c$ [e Å^{-5}]**		ellipticity	
**Bond type / Complex**	**(A)**	**(B)**	**(A)**	**(B)**	**(A)**	**(B)**	**(A)**	**(B)**	**(A)**	**(B)**
Cu1-N1	1.9723	2.0007	0.9628	0.9649	0.622	0.617	9.593	7.803	0.033	0.043
Cu1-N2	1.9603	2.0007	0.9567	0.9649	0.639	0.617	9.890	7.803	0.054	0.043
Cu1-S1	2.2495	2.3280	1.0295	1.0494	0.526	0.482	5.135	3.533	0.026	0.013
Cu1-S2	2.3347	2.3282	1.0645	1.0494	0.445	0.481	4.265	3.530	0.014	0.013
N1-C1 / N1-C8 benz	1.4097	1.4304	0.8606	0.8806	1.943	1.857	-19.404	-17.808	0.054	0.040
N1-C15 / N1-C3 CH	1.2695	1.2805	0.8109	0.8164	2.571	2.531	-20.341	-20.481	0.217	0.183
C15-C16 / C3-C2	1.4590	1.4515	0.7170	0.6994	1.904	1.958	-20.076	-21.421	0.108	0.102
C16-C17 / C2-C1	1.4065	1.4050	0.7096	0.7195	2.052	2.075	-22.199	-22.688	0.191	0.198
C17-S1 / C1-S1	1.7945	1.7878	0.9333	0.9501	1.235	1.263	-6.287	-6.699	0.091	0.140
C1-C2 / C8-C13	1.3987	1.3955	0.7147	0.6751	2.108	2.135	-23.599	-24.122	0.229	0.238
C2-C3 / C13-C13	1.4849	1.4781	0.7431	0.7390	1.793	1.841	-17.640	-18.717	0.029	0.041
C3-C4 / C13-C8	1.3979	1.3955	0.7150	0.7203	2.110	2.135	-23.616	-24.122	0.229	0.238
C4-N2 / C8-N1	1.4099	1.4304	0.8616	0.8806	1.943	1.857	-19.462	-17.808	0.049	0.040
C1-C7 / C8-C9	1.3750	1.3879	0.6777	0.7229	2.195	2.165	-25.785	-24.869	0.196	0.230
C7-C8 / C9-C10	1.3871	1.3828	0.7159	0.6757	2.152	2.184	-24.604	-25.568	0.223	0.189
C8-C9 / C10-C11	1.3763	1.3829	0.6855	0.6894	2.195	2.193	-25.857	-25.809	0.189	0.183
C9-C10 / C11-C12	1.3774	1.3851	0.6940	0.6996	2.191	2.183	-25.785	-25.616	0.190	0.180
C10-C2 / C12-C13	1.3870	1.3892	0.6836	0.7104	2.144	2.154	-24.243	-24.556	0.206	0.203
C3-C11 / C13-C12	1.3863	1.3892	0.7034	0.6788	2.146	2.154	-24.315	-24.556	0.207	0.203
C11-C12 / C12-C11	1.3780	1.3851	0.6947	0.6855	2.188	2.183	-25.689	-25.616	0.190	0.180
C12-C13 / C11-C10	1.3765	1.3829	0.6854	0.6934	2.194	2.193	-25.809	-25.809	0.190	0.183
C13-C14 / C10-C9	1.3758	1.3828	0.6783	0.7070	2.192	2.184	-25.713	-25.568	0.196	0.189
C14-C4 / C9-C8	1.3851	1.3879	0.7165	0.6650	2.159	2.165	-24.749	-24.869	0.225	0.230
C17-C18 / C1-C4	1.3819	1.3812	0.7075	0.7246	2.169	2.173	-24.797	-24.869	0.204	0.213
C18-C19 / C4-C5	1.3775	1.3878	0.6945	0.7053	2.190	2.164	-25.761	-25.158	0.183	0.172

C19-C20 / C5-C6	1.3765	1.3822	0.6920	0.6984	2.201	2.202	-26.098	-26.146	0.180	0.174
C20-C21 / C6-C7	1.3726	1.3811	0.6760	0.6750	2.217	2.204	-26.412	-26.171	0.189	0.178
C21-C16 / C7-C2	1.3953	1.3940	0.7042	0.7120	2.120	2.139	-23.814	-24.315	0.180	0.184
N2-C22 CH	1.2683	1.2805	0.8107	0.8163	2.573	2.531	-20.069	-20.479	0.219	0.183
C22-C23	1.4588	1.4515	0.7156	0.6994	1.907	1.958	-20.168	-21.421	0.106	0.102
C23-C24	1.4058	1.4050	0.7101	0.7195	2.054	2.075	-22.231	-22.688	0.192	0.198
C24-S2	1.7911	1.8320	0.9304	0.9854	1.241	1.165	-6.362	-5.280	0.116	0.078
C24-C25	1.3814	1.3812	0.7087	0.7246	2.169	2.173	-24.797	-24.869	0.204	0.213
C25-C26	1.3779	1.3878	0.6945	0.7053	2.186	2.164	-25.640	-25.158	0.185	0.172
C26-C27	1.3767	1.3822	0.6929	0.6984	2.199	2.202	-26.026	-26.146	0.181	0.174
C27-C28	1.3730	1.3811	0.6756	0.6750	2.214	2.204	-26.315	-26.171	0.190	0.178
C28-C23	1.3937	1.3940	0.6902	0.6820	2.124	2.139	-23.908	-24.315	0.183	0.184
S1-C5 / S1-C14 most	1.8284	1.8320	0.9705	0.9854	1.149	1.165	-4.933	-5.280	0.078	0.078
S2-C6	1.8244	1.7879	0.9668	0.9501	1.157	1.263	-5.041	-6.699	0.082	0.140
C5-C6 / C14-C14	1.5053	1.5106	0.7530	0.7553	1.715	1.704	-15.606	-15.305	0.016	0.006

Table S3 Comparison of SHELXL refinement (**B**)

Compound (**B**)	all data	data trimmed at d_{min} = 0.70 Å
2Θ range for data collection/°	3.232 to 88.884	3.232 to 47.228
Index ranges	$-28 \leq h \leq 28$, $-14 \leq k \leq 28$, $-96 \leq l \leq 97$	$-16 \leq h \leq 16$, $-14 \leq k \leq 16$, $-55 \leq l \leq 53$
Reflections collected	182436	4 096
Independent reflections	20608 [R_{int} = 0.1508, R_{sigma} = 0.2086]	4091 [Rint = 0.0393, Rsigma = 0.0220]
Data/restraints/parameters	20608/0/235	4091/0/235
Goodness-of-fit on F^2	0.725	1.021
Final R indexes [I>=2σ (I)]	R_1 = 0.0380, wR_2 = 0.0757	R1 = 0.0274, wR2 = 0.0673
Final R indexes [all data]	R_1 = 0.2013, wR_2 = 0.0949	R1 = 0.0407, wR2 = 0.0743
Largest diff. peak/hole / e Å^{-3}	0.74/-1.37	0.37/-0.52

Table S4 Selected experimental topological properties associated with bond critical points of complex (**B**) (R_{ij} – the bond path length, d_1 – the first atom-to-BCP distance, ρ_c – BCP electron density, $\nabla^2\rho_c$ – BCP Laplacian).

Bond type	R_{ij} [Å]	d_1 [Å]	ρ_c [e Å^{-3}]	$\nabla^2\rho_c$ [e Å^{-5}]	ellipticity
Cu1-N1[a]	1.9947	0.9903	0.611(7)	10.604(8)	0.04
Cu1-N1[b]	1.9902	0.9659	0.604(7)	10.698(10)	0.08
Cu1-S1[a]	2.2900	1.0393	0.517(5)	6.017(4)	0.08
Cu1-S1[b]	2.2926	1.0452	0.452(6)	6.104(5)	0.05
Cu1-F1[a]	2.5293	1.2948	0.131(1)	2.633(3)	0.05
Cu1-F1[b]	2.5234	1.2737	0.123(1)	2.508(3)	0.08
N1-C8[a]	1.4388	0.7828	1.85(3)	-12.11(9)	0.07
N1-C8[b]	1.4211	0.7439	2.13(4)	-19.9(1)	0.12
C8-C9[a]	1.4048	0.5346	2.18(6)	-26.4 (2)	0.32
C8-C9[b]	1.4003	0.6136	2.25(5)	-28.0 (2)	0.23
C9-C10[a]	1.3986	0.5609	2.09(5)	-22.6 (2)	0.44
C9-C10[b]	1.4077	0.8991	2.01(8)	-33.8 (3)	0.32
C10-C11[a]	1.3932	0.6746	1.76(5)	-10.2 (1)	0.13
C10-C11[b]	1.4079	0.8490	1.86(7)	-8.3 (2)	0.35
C11-C12[a]	1.3789	0.6032	1.64(5)	-4.3 (2)	0.08
C11-C12[b]	1.3796	0.6101	1.74(6)	-3.0 (2)	0.65
C12-C13[a]	1.4020	0.6654	2.08(4)	-17.8 (1)	0.20
C12-C13[b]	1.4672	0.5824	1.50(6)	-9.5 (2)	1.62
C13-C8[a]	1.4092	0.8563	2.08(5)	-25.1 (2)	0.26
C13-C8[b]	1.4718	0.9702	1.68(8)	-22.2 (3)	0.90

a) Experimental refinement on the full data set, b) Experimental refinement on the data set with sin θ/λ < 0.7 Å-1.

[1] Abrahams, S. C. & Keve, E. T. (1971). *Acta Cryst*. **A27**, 157-165.

[2] Meindl, K. & Henn, J. (2008). *Acta Cryst.* A**64**, 404-418.
